# Bis­(benzene­thiol­ato)(2,2′-biquinoline)zinc(II)

**DOI:** 10.1107/S1600536809041336

**Published:** 2009-10-23

**Authors:** Thomas Blake Monroe, Anita Ikonen, Jonathon A. Maner, Daniel S. Jones, Durwin R. Striplin

**Affiliations:** a Department of Chemistry, The University of North Carolina at Charlotte, 9201 University City Blvd, Charlotte, NC 28223, USA; b Department of Chemistry, Davidson College, Davidson, NC 28035, USA

## Abstract

The title compound, [Zn(C_6_H_5_S)_2_(C_18_H_12_N_2_)], was prepared as a model for future complexes that will be incorporated into light-harvesting arrays. The Zn^II^ atom lies on a twofold rotation axis and the ligands are arranged tetra­hedrally around this atom. The benzene­thiol­ate ligand and the biquinoline ligand are nearly perpendicular to one another, making a dihedral angle of 84.09 (5)°. The biquinoline ligand is nearly planar, with a maximum deviation of 0.055 (3) Å from the mean plane of the ring system. In the crystal, the mol­ecules pack in a manner such that the biquinoline ligands are parallel to one another, with a π–π inter­action [interplanar distance = 3.38 (1) Å] with the neighboring biquinoline ligand.

## Related literature

For luminescent complexes of zinc(II), see: Koester (1975 ▶); Crosby *et al.* (1985 ▶); Highland *et al.* (1986 ▶). For related structures, see: Halvorsen *et al.* (1995 ▶); Anjali *et al.* (1999 ▶). For a study of π–π inter­actions involving quinoline ring systems, see: Janiak (2000 ▶). For details of the Cambridge Crystal Structure Database, see: Allen *et al.* (2002 ▶).
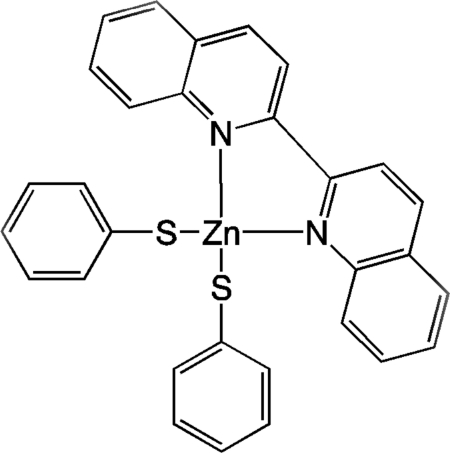


## Experimental

### 

#### Crystal data

[Zn(C_6_H_5_S)_2_(C_18_H_12_N_2_)]
*M* *_r_* = 539.99
Monoclinic, 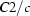

*a* = 17.141 (2) Å
*b* = 11.5591 (8) Å
*c* = 12.8318 (14) Å
β = 93.811 (10)°
*V* = 2536.8 (4) Å^3^
*Z* = 4
Cu *K*α radiation
μ = 3.04 mm^−1^
*T* = 295 K
0.30 × 0.19 × 0.11 mm

#### Data collection

Enraf–Nonius CAD-4 diffractometer
Absorption correction: analytical (Alcock, 1970 ▶) *T*_min_ = 0.531, *T*_max_ = 0.796
4580 measured reflections
2294 independent reflections
1821 reflections with *I* > 2σ(*I*)
*R*_int_ = 0.025
3 standard reflections every 195 reflections intensity decay: 6%

#### Refinement

*R*[*F* ^2^ > 2σ(*F* ^2^)] = 0.027
*wR*(*F* ^2^) = 0.076
*S* = 1.02
2294 reflections
160 parameters
H-atom parameters constrained
Δρ_max_ = 0.25 e Å^−3^
Δρ_min_ = −0.21 e Å^−3^

### 

Data collection: *CAD-4 EXPRESS* (Enraf–Nonius, 1994 ▶); cell refinement: *CAD-4 EXPRESS*; data reduction: *XCAD4* (Harms & Wocadlo, 1995 ▶); program(s) used to solve structure: *DIRDIF* (Beurskens *et al.*, 1999 ▶); program(s) used to refine structure: *SHELXL97* (Sheldrick, 2008 ▶); molecular graphics: *ORTEP-3 for Windows* (Farrugia, 1997 ▶) and *Mercury* (Macrae *et al.*, 2006 ▶); software used to prepare material for publication: *WinGX* (Farrugia, 1999 ▶).

## Supplementary Material

Crystal structure: contains datablocks global, I. DOI: 10.1107/S1600536809041336/su2141sup1.cif

Structure factors: contains datablocks I. DOI: 10.1107/S1600536809041336/su2141Isup2.hkl

Additional supplementary materials:  crystallographic information; 3D view; checkCIF report

## Figures and Tables

**Fig. 1.**
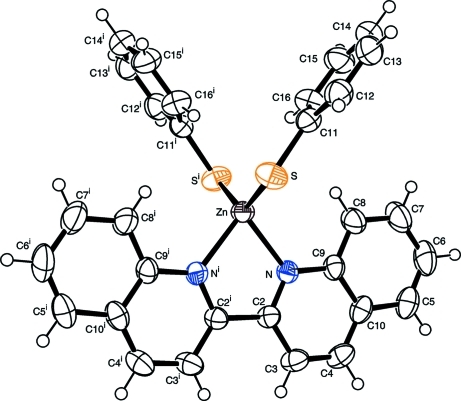
View of the molecular structure of the title compound, with 50% probability displacement ellipsoids [Symmetry code: (i) -x, y, -z + 3/2]

**Fig. 2.**
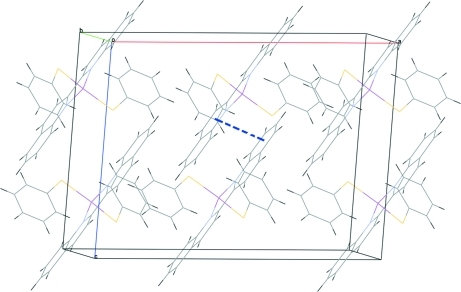
Crystal packing diagram of the title compound, showing the π-π interactions between the biquinoline ligands.

## supplementary crystallographic information

### Comment

Deeply colored, luminescent complexes of zinc(II) arise when this d^10^ ion is coordinated with mixed ligands of benzenethiol anions and dinitrogenpolypyridyl ligands (Koester, 1975; Crosby *et al.*, 1985; Highland *et al.*, 1986). In many cases, the lowest-lying excited state of this class of molecules has been assigned to a charge transfer from the thiol to a π^*^ molecular orbital on the nitrogen heterocycle (Koester, 1975; Highland *et al.*, 1986). The straightforward synthesis of the complexes, the cost, and the variety of ligand substitutions that are possible, enable numerous ways to tune the energy of this ligand-to-ligand charge transfer state. The closed-shell zinc(II) complex of this study absorbs strongly in the visible region of the spectrum and serves as a model for future complexes that will be incorporated into light-harvesting arrays.

The molecular structure of the title compound is illustrated in Fig. 1. The ligands are arranged tetrahedrally around the zinc atom, which lies on a 2-fold rotation axis. The benzenethiolate ligand and the biquinoline ligand are nearly perpendicular to one another, making a dihedral angle of 84.09 (5)°. The benzenethiolate ligands make a 72.30 (5)° angle with one another. The biquinoline ligand is nearly planar, with a maximum deviation of 0.055 (3) Å from the mean plane of the ring system.

In the crystal of the title compound the molecules pack in a manner such that the biquinoline ligands of all molecules are parallel. An exhaustive study has been made (Janiak, 2000) of structures in the Cambridge Structural Database (Allen, 2002) which show π-π interactions between quinoline ring systems. This study showed that parallel ring systems which interact are offset by an amount related to the distance between ring centroids. In the present study, the planes of the quinoline rings related by π-π interactions are ca. 3.38 Å apart. The centeroids of the pyridine ring and the benzene ring are ca. 3.68 Å apart, and the centroid-centroid line makes an angle of 23.3° with the normal to the plane of the quinoline rings. These values are in agreement with those found in the Janiak study. The π-π interactions may account for the near-planarity of the biquinoline ligand.

A search of the Cambridge Structural Database [CSD Version 5.30; Allen, 2002] yielded two chemically comparable structures: bis(Benzenethiolato)-(2,2'-bipyridine-N,*N*')-zinc (Anjali *et al.*, 1999) and (1,2-Benzenedithiolato-S,*S*')-(2,2'-biquinolinato-N,*N*')-zinc(II) (Halvorsen *et al.*, 1995).

### Experimental

The complex was synthesized *via* a general procedure (Crosby *et al.*, 1985). Zn(OAc)_2_.2H_2_O (0.0566 g) was dissolved in 5 ml absolute ethanol and heated. benzenethiol (0.0582 g) was dissolved in 4 ml absolute ethanol and heated. The benzenethiol solution was then added dropwise to the zinc(II) solution with vigorous stirring at reflux. 2,2'-biquinoline (0.0640 g) dissolved in 5 ml absolute ethanol was then slowly added to the refluxing solution. The solution turned orange and was allowed to sit overnight. An orange crystalline solid (0.0848 g) was collected in 47% yield *via* vacuum filtration and washed with cold ethanol. The compound was characterized by ^1^H NMR, UV-VIS absorption spectroscopy, and room temperature and 77 K emission spectroscopy.

### Refinement

The H-atoms were included in calculated positions and treated as riding atoms: C—H = 0.93 Å, with *U*_iso_(H) = 1.2*U*_eq_(parent C-atom).

### Crystal data

**Table:**

[Zn(C_6_H_5_S)_2_(C_18_H_12_N_2_)]	*F*(000) = 1112
*M**_r_* = 539.99	*D*_x_ = 1.414 Mg m^−^^3^
Monoclinic, *C*2/*c*	Cu *K*α radiation, λ = 1.54184 Å
Hall symbol: -C 2yc	Cell parameters from 25 reflections
*a* = 17.141 (2) Å	θ = 9.8–42.1°
*b* = 11.5591 (8) Å	µ = 3.04 mm^−^^1^
*c* = 12.8318 (14) Å	*T* = 295 K
β = 93.811 (10)°	Prism, orange
*V* = 2536.8 (4) Å^3^	0.30 × 0.19 × 0.11 mm
*Z* = 4	

### Data collection

**Table:**

Enraf–Nonius CAD-4 diffractometer	*R*_int_ = 0.025
Non–profiled ω/2θ scans	θ_max_ = 67.5°, θ_min_ = 4.6°
Absorption correction: analytical (Alcock, 1970)	*h* = −20→20
*T*_min_ = 0.531, *T*_max_ = 0.796	*k* = −13→0
4580 measured reflections	*l* = −15→15
2294 independent reflections	3 standard reflections every 195 reflections
1821 reflections with *I* > 2σ(*I*)	intensity decay: 6%

### Refinement

**Table:**

Refinement on *F*^2^	H-atom parameters constrained
Least-squares matrix: full	*w* = 1/[σ^2^(*F*_o_^2^) + (0.0413*P*)^2^ + 0.5756*P*] where *P* = (*F*_o_^2^ + 2*F*_c_^2^)/3
*R*[*F*^2^ > 2σ(*F*^2^)] = 0.027	(Δ/σ)_max_ < 0.001
*wR*(*F*^2^) = 0.076	Δρ_max_ = 0.25 e Å^−^^3^
*S* = 1.02	Δρ_min_ = −0.21 e Å^−^^3^
2294 reflections	Extinction correction: *SHELXL97* (Sheldrick, 2008), Fc^*^=kFc[1+0.001xFc^2^λ^3^/sin(2θ)]^-1/4^
160 parameters	Extinction coefficient: 0.00076 (7)
0 restraints	

### Special details

**Table:**

Geometry. All e.s.d.'s (except the e.s.d. in the dihedral angle between two l.s. planes) are estimated using the full covariance matrix. The cell e.s.d.'s are taken into account individually in the estimation of e.s.d.'s in distances, angles and torsion angles; correlations between e.s.d.'s in cell parameters are only used when they are defined by crystal symmetry. An approximate (isotropic) treatment of cell e.s.d.'s is used for estimating e.s.d.'s involving l.s. planes.

### Fractional atomic coordinates and isotropic or equivalent isotropic displacement parameters (Å^2^)

**Table:**

	*x*	*y*	*z*	*U*_iso_*/*U*_eq_	
Zn1	0.0000	0.23564 (3)	0.7500	0.03902 (14)	
S1	0.10148 (4)	0.31521 (5)	0.84622 (4)	0.05310 (18)	
C2	0.02308 (12)	−0.00711 (17)	0.70235 (14)	0.0373 (4)	
N1	0.03843 (10)	0.09607 (14)	0.66414 (12)	0.0377 (4)	
C11	0.13310 (13)	0.43928 (18)	0.78028 (16)	0.0431 (5)	
C8	0.09842 (15)	0.2144 (2)	0.53830 (18)	0.0564 (6)	
H8	0.0808	0.2807	0.5703	0.068*	
C7	0.14180 (17)	0.2230 (3)	0.4526 (2)	0.0700 (8)	
H7	0.1534	0.2957	0.4267	0.084*	
C10	0.10770 (12)	0.0041 (2)	0.52807 (16)	0.0453 (5)	
C9	0.08090 (12)	0.10452 (19)	0.57723 (15)	0.0409 (5)	
C3	0.04840 (14)	−0.10960 (19)	0.65728 (17)	0.0487 (5)	
H3	0.0371	−0.1810	0.6861	0.058*	
C6	0.16875 (17)	0.1242 (3)	0.4038 (2)	0.0702 (8)	
H6	0.1985	0.1318	0.3462	0.084*	
C4	0.09000 (14)	−0.1033 (2)	0.57026 (17)	0.0514 (5)	
H4	0.1066	−0.1708	0.5390	0.062*	
C13	0.23087 (15)	0.5875 (2)	0.7683 (2)	0.0638 (7)	
H13	0.2775	0.6210	0.7942	0.077*	
C15	0.12222 (16)	0.5836 (2)	0.6443 (2)	0.0609 (7)	
H15	0.0951	0.6146	0.5856	0.073*	
C14	0.19151 (16)	0.6336 (2)	0.6820 (2)	0.0640 (7)	
H14	0.2112	0.6980	0.6492	0.077*	
C16	0.09275 (15)	0.4876 (2)	0.69300 (18)	0.0526 (6)	
H16	0.0457	0.4551	0.6673	0.063*	
C5	0.15209 (15)	0.0177 (3)	0.43955 (18)	0.0589 (6)	
H5	0.1699	−0.0474	0.4057	0.071*	
C12	0.20215 (14)	0.4915 (2)	0.8175 (2)	0.0551 (6)	
H12	0.2296	0.4616	0.8764	0.066*	

### Atomic displacement parameters (Å^2^)

**Table:**

	*U*^11^	*U*^22^	*U*^33^	*U*^12^	*U*^13^	*U*^23^
Zn1	0.0495 (2)	0.0321 (2)	0.0360 (2)	0.000	0.00750 (15)	0.000
S1	0.0643 (4)	0.0479 (3)	0.0456 (3)	−0.0058 (2)	−0.0081 (3)	0.0087 (2)
C2	0.0443 (10)	0.0359 (10)	0.0317 (9)	0.0018 (8)	0.0014 (8)	−0.0010 (8)
N1	0.0427 (9)	0.0378 (8)	0.0332 (8)	−0.0015 (7)	0.0070 (7)	−0.0011 (7)
C11	0.0473 (11)	0.0395 (11)	0.0428 (11)	0.0013 (9)	0.0053 (9)	−0.0050 (9)
C8	0.0727 (15)	0.0512 (13)	0.0478 (12)	−0.0120 (12)	0.0220 (11)	−0.0036 (11)
C7	0.0817 (18)	0.0749 (18)	0.0565 (15)	−0.0219 (15)	0.0276 (13)	0.0013 (14)
C10	0.0416 (11)	0.0590 (13)	0.0350 (10)	0.0065 (10)	0.0014 (8)	−0.0100 (9)
C9	0.0427 (11)	0.0487 (11)	0.0319 (9)	−0.0024 (9)	0.0065 (8)	−0.0053 (8)
C3	0.0673 (14)	0.0376 (10)	0.0411 (11)	0.0069 (10)	0.0031 (10)	−0.0029 (9)
C6	0.0652 (16)	0.100 (2)	0.0484 (14)	−0.0113 (15)	0.0260 (12)	−0.0077 (14)
C4	0.0640 (14)	0.0471 (12)	0.0430 (12)	0.0137 (11)	0.0027 (10)	−0.0090 (10)
C13	0.0458 (13)	0.0585 (15)	0.088 (2)	−0.0082 (11)	0.0126 (13)	−0.0126 (14)
C15	0.0744 (17)	0.0461 (12)	0.0626 (16)	−0.0010 (12)	0.0081 (13)	0.0118 (12)
C14	0.0654 (16)	0.0470 (14)	0.0823 (18)	−0.0036 (12)	0.0257 (14)	0.0036 (13)
C16	0.0613 (14)	0.0447 (12)	0.0508 (12)	−0.0067 (10)	−0.0024 (11)	0.0027 (10)
C5	0.0553 (14)	0.0800 (17)	0.0426 (12)	0.0063 (13)	0.0132 (10)	−0.0136 (12)
C12	0.0485 (13)	0.0562 (14)	0.0603 (14)	0.0008 (11)	0.0020 (11)	−0.0058 (12)

### Geometric parameters (Å, °)

**Table:**

Zn1—N1^i^	2.0849 (16)	C10—C9	1.412 (3)
Zn1—N1	2.0849 (16)	C10—C5	1.417 (3)
Zn1—S1	2.2607 (6)	C3—C4	1.366 (3)
Zn1—S1^i^	2.2607 (6)	C3—H3	0.9300
S1—C11	1.768 (2)	C6—C5	1.351 (4)
C2—N1	1.323 (3)	C6—H6	0.9300
C2—C3	1.400 (3)	C4—H4	0.9300
C2—C2^i^	1.500 (4)	C13—C14	1.366 (4)
N1—C9	1.375 (2)	C13—C12	1.383 (4)
C11—C12	1.385 (3)	C13—H13	0.9300
C11—C16	1.393 (3)	C15—C14	1.380 (4)
C8—C7	1.371 (3)	C15—C16	1.386 (3)
C8—C9	1.405 (3)	C15—H15	0.9300
C8—H8	0.9300	C14—H14	0.9300
C7—C6	1.396 (4)	C16—H16	0.9300
C7—H7	0.9300	C5—H5	0.9300
C10—C4	1.396 (3)	C12—H12	0.9300
			
N1^i^—Zn1—N1	78.61 (9)	C4—C3—C2	119.0 (2)
N1^i^—Zn1—S1	106.55 (5)	C4—C3—H3	120.5
N1—Zn1—S1	110.17 (5)	C2—C3—H3	120.5
N1^i^—Zn1—S1^i^	110.17 (5)	C5—C6—C7	120.7 (2)
N1—Zn1—S1^i^	106.55 (5)	C5—C6—H6	119.7
S1—Zn1—S1^i^	131.98 (3)	C7—C6—H6	119.7
C11—S1—Zn1	108.53 (7)	C3—C4—C10	120.2 (2)
N1—C2—C3	122.36 (18)	C3—C4—H4	119.9
N1—C2—C2^i^	115.52 (11)	C10—C4—H4	119.9
C3—C2—C2^i^	122.11 (12)	C14—C13—C12	120.8 (2)
C2—N1—C9	119.58 (17)	C14—C13—H13	119.6
C2—N1—Zn1	115.11 (12)	C12—C13—H13	119.6
C9—N1—Zn1	125.20 (14)	C14—C15—C16	120.5 (2)
C12—C11—C16	118.0 (2)	C14—C15—H15	119.7
C12—C11—S1	118.08 (17)	C16—C15—H15	119.7
C16—C11—S1	123.91 (17)	C13—C14—C15	119.2 (2)
C7—C8—C9	119.4 (2)	C13—C14—H14	120.4
C7—C8—H8	120.3	C15—C14—H14	120.4
C9—C8—H8	120.3	C15—C16—C11	120.5 (2)
C8—C7—C6	120.9 (3)	C15—C16—H16	119.8
C8—C7—H7	119.5	C11—C16—H16	119.8
C6—C7—H7	119.5	C6—C5—C10	120.6 (2)
C4—C10—C9	118.17 (19)	C6—C5—H5	119.7
C4—C10—C5	123.5 (2)	C10—C5—H5	119.7
C9—C10—C5	118.4 (2)	C13—C12—C11	120.9 (2)
N1—C9—C8	119.33 (19)	C13—C12—H12	119.5
N1—C9—C10	120.6 (2)	C11—C12—H12	119.5
C8—C9—C10	120.04 (19)		

Symmetry codes: (i) −*x*, *y*, −*z*+3/2.
